# Neuroprotection of Grape Seed Extract and Pyridoxine against Triton-Induced Neurotoxicity

**DOI:** 10.1155/2016/8679506

**Published:** 2016-05-16

**Authors:** Heba M. Abdou, Mayssaa M. Wahby

**Affiliations:** ^1^Faculty of Science, Zoology Department, Alexandria University, Alexandria 21311, Egypt; ^2^Faculty of Science, Biochemistry Department, Alexandria University, Alexandria 21311, Egypt

## Abstract

Triton WR-1339 administration causes neurotoxicity. Natural products and herbal extracts can attenuate cerebral injury. In the present study, we investigated the neuroprotective role of grape seed extract and/or vitamin B6 against triton-induced neurotoxicity. Thirty-five adult male albino rats of the Sprague-Dawley strain, weighing 140–145 g, were divided into five groups: control, triton, grape seed extract + triton, grape seed extract + triton + vitamin B6, and vitamin B6 + triton. The hematological and biochemical analyses were carried out. Alteration in iNOS mRNA gene expression was determined using reverse-transcriptase PCR analysis. In addition, qualitative DNA fragmentation was examined using agarose gel electrophoresis. Triton-treatment caused significant disturbances in the hematological parameters, the neurological functions, and the antioxidant profile. Also, triton significantly increased the iNOS mRNA expression and DNA damage. Our results showed that grape seed extract and/or vitamin B6 could attenuate all the examined parameters. These natural substances could exhibit protective effects against triton-induced neurological damage because of their antioxidative and antiapoptotic capacities.

## 1. Introduction

The redox imbalance, inflammation, and apoptosis are the major mechanisms of cerebral injury [1, 2]. The increase of oxidative metabolic activity in addition to the low concentrations of endogenous antioxidants causes brain oxidative insults [3]. The administration of the nonionic detergent, triton WR1339 (Tyloxapol), was able to increase the oxidative markers in animals. This oxidation plays a crucial role in the pathologic processes [4]. Experimentally, natural products and herbal extracts can attenuate cerebral injury and showed protective effects against brain damage [5, 6].

Grapes (*Vitis vinifera*) are among the fruits that have the highest phenolic compound contents. It has been associated with the antioxidant, anti-inflammatory, anticarcinogenic, and antibacterial activities [7]. Grape seed extract (GSE) contains plant flavonoids such as proanthocyanidins which are potent antioxidants and exert many health-promoting effects [8]. The antioxidant activities of procyanidins are exerted directly by scavenging reactive oxygen species (ROS) as well as by chelating redox-active transition metals, such as iron and copper [9]. Previous studies have suggested that wild grape root and stem have antiangiogenic, antioxidant, anti-inflammatory, and neuroprotective effects [10].

Vitamin B6 is a generic term that refers to the six interconvertible pyridine compounds (vitamers): pyridoxine (PN, commonly known as vitamin B6), pyridoxamine (PM), pyridoxal (PL), and their 5′-phosphorylated forms (PNP, PMP, and PLP, resp.) [11]. An additional function of B6 vitamers is to act as reactive oxygen species (ROS) scavengers and as factors able to increase resistance to biotic and abiotic stress. PLP and PN may also function as regulators of membrane ion transporters and have been found to bind to steroid receptors to modulate transcription factors [12]. Pyridoxine (vitamin B6) is a water-soluble vitamin and is present in whole grains, legumes, potatoes, nuts, fish, and poultry. It participates in protein metabolism, amino acid, and monoamine neurotransmitter synthesis [13]. Also, it is involved in the methionine/glutathione transsulfuration pathway to form the natural antioxidant, glutathione. Pyridoxine is converted by all organs of the body to pyridoxal 5-phosphate (PLP) and pyridoxamine, which serve as coenzymes for transaminase reaction [13]. PLP has been reported to reduce the complications associated with coronary artery disease, diabetes, hypertension, aging, and neurodegenerative disorders [14]. In this study, we aimed to investigate the neuroprotective role of GSE, the combination between GSE and B6, and B6 alone against triton (WR-1339)-induced neurotoxicity. This was carried out through the examination of important biomarkers that imply neurological, biochemical, and molecular changes.

## 2. Materials and Methods

### 2.1. Animals

Thirty-five adult male albino rats of the Sprague-Dawley strain, weighing 140–145 g, were purchased from the Faculty of Medicine, Alexandria University, Alexandria, Egypt. Animals were housed 7 per cage and were fed a rodent laboratory chow and water* ad libitum*, kept on 12 h light-dark cycle periods, and acclimatized for at least one week prior to the experiment. The local committee approved the design of the experiments, and the protocol conforms to the guidelines of the National Institutes of Health (NIH).

### 2.2. Experimental Design and Sample Collection

After one week of acclimation, animals were divided into five equal groups, 7 per each. The first group was the control. The second was triton group; rats were injected intraperitoneally (IP) with triton (50 mg/kg BW) day after day for 4 weeks according to Bhuvaneswari and Sasikuma [15]. In GSE + triton-treated group, rats were orally given GSE (300 mg/kg BW) according to Sreemantula et al. [16] daily for 4 weeks plus triton. In the GSE + triton + B6-treated group, rats were orally given triton, GSE, and B6 (300 mg/kg BW, 12 mg/kg BW, and 50 mg/kg BW, resp.). In the B6 + triton-treated group, rats of this group were orally given B6 (12 mg/kg BW) according to Basu and Mann [17] daily for 4 weeks plus triton (50 mg/100 g BW). At the end of the experiment, rats were starved for 12 h and then sacrificed by decapitation under diethyl ether anesthesia. Blood samples were collected in tubes containing heparin for hematological analysis. The brain tissues were immediately removed and kept at −80°C till analysis. The brain tissues were homogenized (10%, w/v), separately, in ice-cold phosphate buffer (0.01 M, pH 7.4) containing 1.15% KCl in a Potter-Elvehjem type homogenizer. The homogenate was centrifuged at 10,000 ×g for 20 min at 4°C and the resultant supernatant was used for the biochemical analysis.

### 2.3. Hematological Examination

The noncoagulated blood samples were tested, shortly after collection, for hemoglobin (Hb), total erythrocyte count (TEC), packed cells volume (PCV), total leukocyte count (TLC), and platelets count by Particle Counter (ERMA Inc., Tokyo; Model PCE-210).

### 2.4. Biochemical Parameters

The level of serotonin in the brain was determined by the spectrofluorimetry according to Schlumpf et al. [18]. Acetyl cholinesterase (AChE; EC 3.1.1.7) activity in the brain was estimated using acetylcholine iodide as a substrate according to the method of Ellman et al. [19]. Tissue supernatant thiobarbituric acid-reactive substances (TBARS) were measured at 532 nm using 2-thiobarbituric acid (2,6-dihydroxypyrimidine-2-thiol, TBA) and extinction coefficient of 156,000 M^−1^ cm^−1^ was used for calculation [20]. Reduced glutathione (GSH) content was assayed using 5,5-dithiobis(2-nitrobenzoic acid, DTNB) for color development and its density was measured at 412 nm [21]. The catalase (CAT; EC 1.11.1.6) converts H_2_O_2_ into water and its activity in tissue supernatant was measured spectrophotometrically at 240 nm [22].

Superoxide dismutase (SOD; EC 1.15.1.1) activity was estimated according to Misra and Fridovich [23]. The activity of glutathione peroxidase (GPX) was determined using the method of Chiu et al. [24] in the brain extracts. The activity of lactate dehydrogenase (LDH) was determined in the tissue supernatant according to Moss et al. [25]. The protein content of the brain tissue was determined by the method of Lowry et al. [26] using bovine serum albumin as a standard.

### 2.5. RT-PCR for iNOS Messenger RNA Gene Expression

Brain tissues were kept in RNA later, a reagent for immediate stabilization of the gene expression profile in harvested animal tissues. Then, total RNA was extracted from brain tissues according to Chomczynski and Sacchi [27] using Biozol RNA Isolation Kit. The total RNA extract was resuspended in 50–100 *μ*L RNase-free H_2_O. RNA concentration was determined using the following equation: 1 absorbance unit at 260 nm corresponds to approximate concentration of 40 *μ*g/mL of single-stranded RNA. The purity of the RNA preparation was estimated to be 1.8–2.0 according to the ratio of absorbance readings at 260 nm and 280 nm [Abs 260/Abs 280] [27]. Alteration in iNOS mRNA gene expression level was determined using reverse-transcriptase PCR analysis. One-step RT-PCR (RT/PCR Master Mix Gold Beads, BIORON) reaction was used for the cDNA synthesis and for amplification of target gene using specific primer sets as follows.

Primers for iNOS (GCTGCCAGGGTCACAACTTT and CCAGTGACACTGTGTCCCGT) and for beta actin (GCTTCTTTGCAGCTCCTTCGT and CGTCATCCATGGCGAACTG) yielded PCR products of 71 and 59 bp, respectively. Specificity of PCR was checked by analyzing the melting curve. Relative mRNA levels were determined by comparing (a) the PCR cycle threshold between cDNA of iNOS and beta actin and (b) values between treated and untreated conditions as described previously [28]. The amplified RT-PCR product (10 *μ*L) was mixed with 2 *μ*L of sample loading dye, electrophoresed on 1.5% agarose gel (1.5 g/100 mL 0.5x TBE) containing 10 *μ*g/mL ethidium bromide (EtBr) dye, and visualized with gel documentation system [29].

### 2.6. Qualitative DNA Fragmentation Assay by Agarose Gel Electrophoresis

DNA was extracted using the genomic DNA purification kit purchased from Bio Basic Inc., Canada. Afterwards, DNA was quantified spectrophotometrically and then loaded onto agarose gel (15 *μ*g/lane). DNA laddering was determined by constant voltage mode electrophoresis at 80 V, for 45 min on a 1.2% agarose gel containing 0.5 *μ*g/mL ethidium bromides. A 1 kbp ladder (Bioron) served as DNA base pair marker. Gels were illuminated with 300 nm UV light and a photographic record was made [30].

### 2.7. Statistical Analysis

Data were analyzed according to Steel and Torrie [31]. Statistical significance of the difference in values of control and treated animals was calculated by (*F*) test at 5% significance level. Data of the present study were statistically analyzed by using LSD Multiple Range Test [32].

## 3. Results

### 3.1. Hematological Examination

The results of triton-treated group showed significant (*p* < 0.05) decrease in the total leukocyte count (TLC), total erythrocyte count (TEC), hemoglobin (Hb) concentration, and the packed cells volume (PCV) while there was significant (*p* < 0.05) increase in the platelet count (PLT) compared to the controls. On the other hand, the hematological parameters in triton + GSE- and/or triton-treated groups were significantly (*p* < 0.05) improved compared to the triton-treated one (Table 1).

### 3.2. Biochemical Parameters

The data represented in Table 2 summarized the biochemical parameters of the experimental groups. The results indicated that the brain serotonin level was significantly (*p* < 0.05) decreased in rats of triton-treated group compared to control. Meanwhile, serotonin level was significantly (*p* < 0.05) increased in groups treated with GSE + triton, GSE + triton + B6, or B6 + triton compared to those treated with triton only. Also, the brain acetyl cholinesterase activity was significantly (*p* < 0.05) increased in triton-treated group compared to that of the control. Meanwhile, treatment with GSE + triton, GSE + triton + B6, or B6 + triton exhibited significant (*p* < 0.05) decrease in acetyl cholinesterase activity when compared to triton-treated group.

Moreover, the brain glutathione (GSH) level as well as the activities of brain glutathione peroxidase (GPX), superoxide dismutase (SOD), catalase (CAT), and lactate dehydrogenase (LDH) was significantly (*p* < 0.05) decreased in triton-treated group compared to the control one (Table 2). Interestingly, all the above parameters showed significant (*p* < 0.05) increases in groups treated with GSE + triton, GSE + triton + B6, or B6 + triton as compared to triton-treated one. Conversely, the level of brain malondialdehyde (MDA) was significantly (*p* < 0.05) increased in triton-treated group compared to that of control. Meanwhile, the MDA levels of GSE + triton-, GSE + triton + B6-, and B6 + triton-treated groups were significantly (*p* < 0.05) decreased in comparison to triton-treated one.

### 3.3. RT-PCR for iNOS Messenger RNA Gene Expression

**Figure 1:**
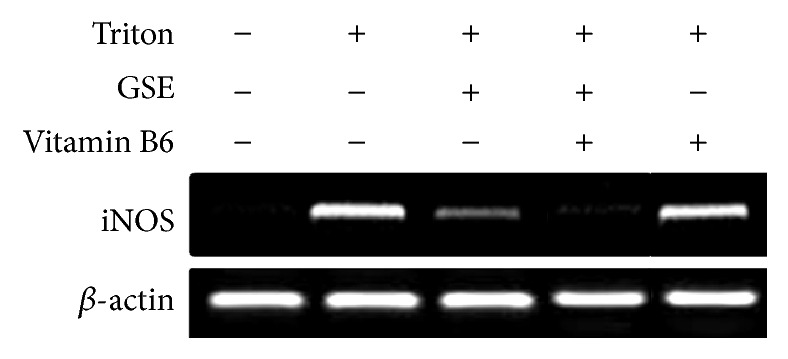
Reverse-transcriptase PCR (RT-PCR) analysis of iNOS mRNA expression in the different experimental groups.

**Figure 2:**
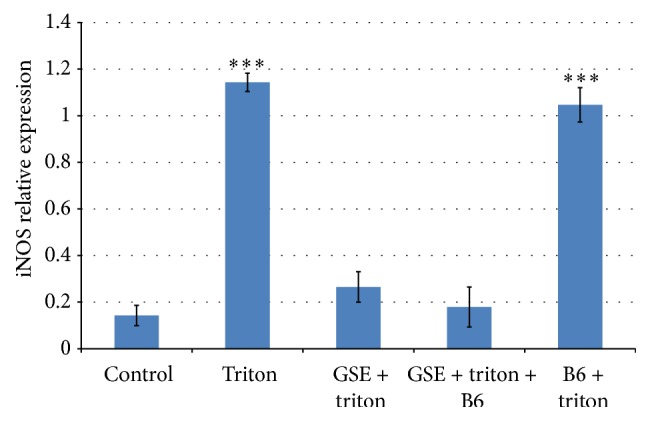
iNOS mRNA expression in the different experimental groups relative to *β* actin level as internal control. ^*∗∗∗*^ *p* < 0.001 calculated with reference to control sample.

The levels of iNOS mRNA expression in brain tissue of control and treated rats were measured by RT-PCR relative to *β*-actin as represented in Figures 1 and 2. Our results indicated that treatment with triton or B6 + triton significantly (^*∗∗∗*^ *p* < 0.001) induced iNOS mRNA expression in brain tissue (lanes 2 and 5) as compared to control (lane 1). However, B6 + triton-treatment caused nonsignificant downregulation in iNOS mRNA expression level (lane 5) compared to triton-treatment (lane 2). On the other hand, the iNOS mRNA expression was barely detectable in both GSE + triton- and the GSE + triton + B6-treated groups (lanes 3 and 4, resp.), the same as the control (lane 1). The treatment with GSE + triton and the GSE + triton + B6 significantly downregulated the iNOS mRNA expression level in comparison to triton-treatment.

### 3.4. Qualitative DNA Fragmentation Assay by Agarose Gel Electrophoresis

**Figure 3:**
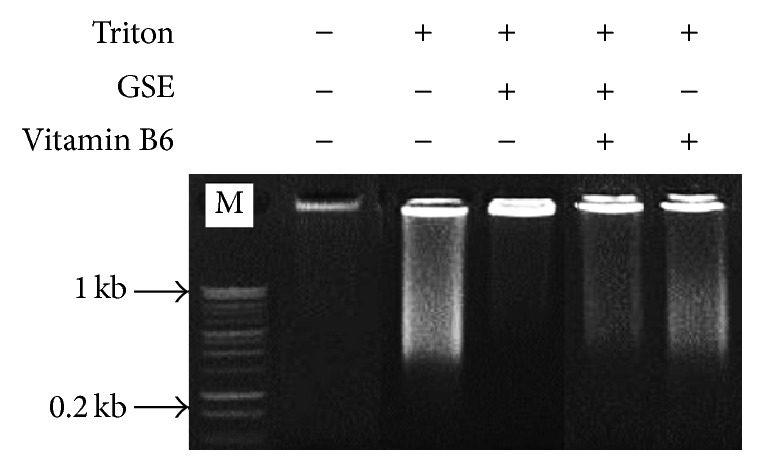
Detection of brain DNA fragmentation on agarose gel electrophoresis of the different experimental groups. The first lane, control; the second lane, triton-treated rats; the third lane, GSE + triton-treated rats; the fourth lane, the GSE + triton + B6-treated rats; the fifth lane, B6 + triton-treated rats.

The DNA fragmentation of rat brain tissue was detected on agarose gel electrophoresis as shown in Figure 3. The DNA fragmentation in triton-treated rats (lane 2) showed smear formation, which may indicate a typical feature of necrosis, compared to the control (lane 1). Interestingly, GSE + triton-treatment (lane 3) prevents the necrotic effect of triton as indicated by the complete absence of smear formation. Also, the treatment with GSE + triton + B6 (lane 4) or B6 + triton (lane 5) could ameliorate the necrotic effect of triton.

## 4. Discussion

The present study was designed to examine the protective effect of grape seed extract or/and vitamin B6 against triton (WR-1339)-induced neurotoxicity.

### 4.1. Hematological Examination

The results represented in Table 1 indicated that the triton-treatment caused significant decreases in the total leukocyte, the total erythrocyte count, the hemoglobin level, and the packed cells volume as compared to the controls. Meanwhile, triton-treatment caused significant increase in platelet count compared to control. The hematological parameters in GSE and/or B6-treated groups were significantly improved compared to the triton-treated one.

### 4.2. Biochemical Parameters

Our results indicated that brain serotonin level was significantly decreased in triton-treated group while the acetyl cholinesterase activity was increased compared to controls. These biochemical parameters were improved in rats treated with GSE + triton, GSE + triton + B6, and B6 + triton compared to triton-treated group (Table 2). Many studies have provided evidence that GSE contains proanthocyanidin which has potent radical scavenging ability and antioxidant properties and thus provides significant neuroprotective effect [33, 34]. Also, Soltaninejad et al. [35] reported that GSE treatment restored AChE activity near to the control value indicating their ameliorating effect. The phenolic dietary antioxidant supplements of grape seeds have been also shown to enhance hippocampal neurogenesis [36]. In addition, Choi et al. [37] found that administration of GSE caused the regulation of brain epinephrine, noradrenaline, serotonin, and dopamine. Plecko et al. [38] indicated that pyridoxal phosphate-dependent enzymes play a role in the biosynthesis of five important neurotransmitters (serotonin, dopamine, adrenaline, noradrenaline, and g-amino butyric acid). Tryptophan is converted to 5-hydroxy tryptophan by an enzyme, tryptophan hydroxylase, and 5-hydroxy tryptophan is converted to 5-hydroxy tryptamine (5-HT) in the presence of tryptophan decarboxylase and the coenzyme pyridoxal phosphate (PLP). So, vitamin B6 supplementation and a tryptophan-rich diet can alleviate major depressive symptoms among patients with multiple drug addiction [39]. Specifically, vitamins B2, B6, and B12 are important for the metabolism of dopamine and noradrenaline within the central nervous system [40].

The brain GSH level as well as GPX, SOD, and CAT activities showed significant decrease, while MDA level significantly increased in triton-treated group when compared to control (Table 2). On the other hand, the treatment with triton in combination with either GSE and/or B6 significantly increased the levels of GSH and the activities of GPX, SOD, and CAT in comparison to triton-treatment only. Meanwhile, the treatment with triton in combination with GSE and/or B6 significantly decreased the MDA level compared to triton-treated group. In accordance with our results, Oh and Lim [41] showed that the level of plasma TBARS was increased 18 h after triton WR-1339 administration to mice and the activity of CAT and GPx was decreased compared with the control group. Alía et al. [42] found that the cellular antioxidant enzyme system, including GR, GPx, CAT, and SOD, plays an essential role in the defense against oxidative stress and can be used as biomarkers for the antioxidant response. Polyphenolic compounds that are present in grape seeds have powerful antioxidant properties and GSE may inhibit lipid peroxidation by scavenging free radicals and increasing intracellular concentration of glutathione [43]. Also, GSE inhibits the enzyme systems that are responsible for the production of free radicals [44]. The administration of grape seed proanthocyanidin treatment could alleviate the brain injury caused by hypoxia from sleep breathing disorder [34]. The chronic hypoxia increased the levels of reactive oxygen species and therefore overloads the endogenous clearing system, like SOD [45]. In addition, B6 is cofactor for cystathionine-synthase (CBS), catalyzing the transsulfuration pathway that is essential for GSH synthesis [46]. Moreover, the SOD activities and the antioxidant potential in kidney tissue of vitamin B6 deficient rats were significantly lower than those of the control ones [47]. In oxidative stress, SOD converts reactive superoxide into less harmful hydrogen peroxide, which is broken down into water and oxygen by CAT and GPx. Also, GPx catalyzes the reduction of lipid hydroperoxides to hydroxides by GSH and GR recycles the oxidized glutathione back to GSH [48]. It is noteworthy that the brain LDH activity exhibited significant decrease in triton-treated group compared to control (Table 2). Meanwhile, treatment with GSE and/or vitamin B6 plus triton caused significant increase in its activity in the brain extract (Table 2). In accordance with our results, Singh et al. [49] found that flavonoids in GSE exert many health-promoting effects, including the ability to increase intercellular antioxidant levels, decrease capillary permeability and fragility, and scavenge oxidants and free radicals. Coinciding with our results, Saada et al. [50] reported that GSE attenuates the ionizing radiation-induced oxidative stress in heart tissues by a significant amelioration of serum lactate dehydrogenase, creatine phosphokinase, and aspartate aminotransferase enzymes activity. They also found that GSE attenuates the oxidative stress in pancreas tissues in rats by significant improvement in hyperglycemia and hyperinsulinemia.

### 4.3. Molecular Analysis

Our results represented in Figure 1 indicated that the treatment with GSE + triton or the GSE + triton + B6 (lanes 3 and 4) significantly downregulated the iNOS mRNA expression and maintains the control level (lane 1). Meanwhile, B6 + triton (lane 5) could downregulate the iNOS mRNA expression level, but in a nonsignificant manner in comparison to triton-treatment. The study of Olivenza et al. [51] indicated that the long-term exposure to stress leads to neurodegenerative changes in many species, including humans. This phenomenon was investigated through the role of endogenously released nitric oxide (NO) and the possible induction of the inducible NO synthase (iNOS) isoform. Ponnuswamy et al. [52] observed that immobilization of adult male rats for 6 h during 21 days (stress condition) increases the activity of a calcium-independent NO synthase and induces the expression of iNOS in cortical neurons as seen by immunohistochemical and western blot analysis. Potential proatherogenic effects of iNOS include iNOS mediated oxidative stress and iNOS expression in different cellular compartments. On the other hand, Terra et al. [53] reported that GSE reduced nitric oxide (NO) overproduction in stimulated macrophages via modulation of iNOS expression. Terra et al. [53] found that the IC50 value of GSE (50 *μ*g/mL) was more potent than that of aspirin (3 *μ*M), indomethacin (20 *μ*M), and dexamethasone (9 nM) as regards suppressing nitric oxide synthesis. Vitamin B6 (pyridoxal) pretreatment of RAW cells inhibited LPS-induced expression of iNOS and COX-2 at the mRNA and protein levels. It inhibited LPS-induced nuclear translocation of the NF-*κ*B, the proinflammatory transcription factor. Furthermore, elevating dietary vitamin B6 suppressed NO production* in vivo* in response to LPS administration. Therefore, the anti-inflammatory effect of vitamin B6 is mediated by suppression of NF-*κ*B activation [54]. The DNA degradation on agarose gel electrophoresis was represented in Figure 3. The observed smear pattern of DNA degradation could indicate a necrotic effect in triton-treated group (lane 2) as compared to the control (lane 1). Otherwise, GSE-treated group (lane 3) showed no evidence of DNA degradation compared to the triton-treated group (lane 2). Also, the treatment with GSE + triton + B6 or B6 + triton (lanes 4 and 5) could reduce the necrotic effect induced by triton (lane 2) but in lesser extent than the treatment with GSE + triton. In further support of our results, the previous studies indicated that triton X-100 efficiently induced the apoptotic cell death in hepatoma cell line [55]. Meanwhile, Mahmood [56] found that GSE is rich in polyphenols and flavonoids scavenging peroxynitrite-induced DNA damage in isolated human lymphocytes. Thereby, it protected DNA from nitrogen species-induced damage. Similarly, GSE demonstrated significant protective ability against oxidative damage in rat leukocytes [57]. GSE (60 mg/kg) also showed neuroprotective effects on neuronal injury induced by transient forebrain ischemia in gerbil achieved by inhibiting DNA damage in the gerbil hippocampus [58]. Furthermore, GSE (100 mg/kg, 30 days) could inhibit the accumulation of age-related oxidative DNA damage in the spinal cord and in various brain regions [59]. The protective role of anthocyanins and their derivatives against oxidative stress, apoptosis, and DNA damage in rat smooth muscle and hepatoma cells induced by tertiary-butyl hydroperoxide was confirmed by the study of Lazzé et al. [60] Polyphenols are important metabolic modulators by virtue of their ability to moderate and influence several cellular processes such as signaling, proliferation, apoptosis, and redox balance [61]. Charvet et al. [62] suggested that pyridoxamine treatment scavenges lipid peroxides in mouse retina, as exemplified by isolevuglandins, and improves retinal mitochondrial morphology after animal exposure to bright light. Pyridoxamine supplementation should be considered for inclusion in antioxidant vitamin formulations. Furthermore, Depeint et al. [63] found that the protective effect of pyridoxal against protein carbonylation and DNA damage was maintained over time, and, in the case of DNA oxidation, pyridoxal exhibited an antidotal or rescue effect.

## Figures and Tables

**Table 1:** Changes in the hematological parameters in the blood samples of the different experimental groups.

Source	Parameters	Experimental groups				
		Control	Triton	GSE + triton	GSE + triton + B6	B6 + triton
Blood	HB	12.68 ± 0.22	6.74^a^ ± 0.28	10.42^ab^ ± 0.14	11.66^ab^ ± 0.07	9.84^ab^ ± 0.093
			−46.8%	−17.9%	−8%	−22%
	PCV	37.99 ± 0.59	22.06^a^ ± 0.41	32.71^ab^ ± 0.50	35.61^ab^ ± 0.22	28.48^ab^ ± 0.51
			−41.9%	−13.9%	−6%	−25%
	TEC	4.70 ± 0.095	2.58^a^ ± 0.06	3.80^ab^ ± 0.05	4.22^ab^ ± 0.12	3.06^ab^ ± 0.07
			−45%	−19%	−10%	−34.9%
	TLC	8.20 ± 0.09	4.94^a^ ± 0.07	5.90^ab^ ± 0.095	8.12^b^ ± 0.10	6.56^ab^ ± 0.17
			−39.7%	−28%	−0.97%	−20%
	PLT	309.60 ± 0.51	552.40^a^ ± 1.36	299.60^ab^ ± 2.23	309.00^b^ ± 0.55	283.00^ab^ ± 3.21
			78.4%	−3%	−0.19%	−8.6%

Values are expressed as means ± SE.

^a^The mean values are significantly different in comparison with the control group at *p* ≤ 0.05.

^b^The mean values are significantly different in comparison with the triton-intoxicated group at *p* ≤ 0.05.

Hb: hemoglobin (g/dL); PCV: packed cells volume (%); TEC: erythrocyte count (×10^12^ L^−1^); TLC: total leukocyte counts (×10^9^ L^−1^); PLT: platelets (×10^12^ L^−1^).

**Table 2:** Changes in the levels of serotonin, GSH, and MDA and the activities of AchE, GPX, SOD, CAT, and LDH in brain extract of different experimental groups.

Parameters	Experimental groups				
	Control	Triton	GSE + triton	GSE + triton + B6	B6 + triton
AchE^A^	21.34 ± 0.33	35.38^a^ ± 0.22	22.91^ab^ ± 0.21	21.28^b^ ± 0.39	23.90^ab^ ± 0.28
		65.8%	7%	−0.28%	12%

Serotonin^B^	350.70 ± 0.61	132.56^a^ ± 0.26	280.88^ab^ ± 2.23	252.52^ab^ ± 0.40	232.22^ab^ ± 0.33
		−62%	−20%	−28%	−33.8%

GSH^C^	50.26 ± 0.24	23.84^a^ ± 0.38	42.14^ab^ ± 0.17	49.76^b^ ± 0.14	43.23^ab^ ± 0.17
		−52.6%	−16%	−1%	−14%

GPX^D^	53.99 ± 0.18	19.24^a^ ± 0.29	41.08^ab^ ± 0.27	56.08^ab^ ± 0.27	45.85^ab^ ± 0.28
		−64%	−24%	3.9%	−15%

SOD^D^	65.64 ± 0.28	26.75^a^ ± 0.13	51.08^ab^ ± 0.26	60.16^ab^ ± 0.32	52.60^ab^ ± 0.25
		−59%	−22%	−0.1%	−20%

CAT^D^	60.55 ± 0.26	16.14^a^ ± 0.41	52.90^ab^ ± 0.19	58.15^ab^ ± 0.35	47.63^ab^ ± 0.37
		−89.9%	−12.6%	−4%	−21%

MDA^E^	24.84 ± 0.11	76.24^a^ ± 0.21	34.60^ab^ ± 0.39	28.44^ab^ ± 0.25	36.86^ab^ ± 0.17
		206.9%	39%	14%	48%

LDH^F^	113.20 ± 0.42	37.69^a^ ± 0.30	95.94^ab^ ± 0.11	101.46^ab^ ± 0.15	93.44^ab^ ± 0.28
		−66.7%	−15%	−10%	−17%

Values are expressed as means ± SE.

^a^The mean values are significantly different in comparison with the control group at *p* ≤ 0.01.

^b^The mean values are significantly different in comparison with the triton-intoxicated group at *p* ≤ 0.01.

A: *μ*mol substrate hydrolyzed/min/mg protein, B: ng/g tissue, C: *μ*mol/g tissue, D: U/mg protein, E: nmol/g tissue, and F: mg/g tissue.
